# Widening Access to Surgical Education Through Free Digital Platforms: An Evaluation of the Mind the Bleep Surgical Team's Impact

**DOI:** 10.7759/cureus.74888

**Published:** 2024-12-01

**Authors:** Siddhant Pherwani, Aditya Gangal, Srutti Suresan, Cyra Asher, Akash Doshi

**Affiliations:** 1 Breast Surgery, Guy’s and St Thomas' NHS Foundation Trust, London, GBR; 2 Internal Medicine, Northwick Park Hospital, London North West University Healthcare NHS Trust, London, GBR; 3 General Surgery, Bradford Teaching Hospitals NHS Foundation Trust, Bradford, GBR; 4 Emergency, Oxford University Hospitals NHS Foundation Trust, Oxford, GBR; 5 Diabetes and Endocrinology, Royal London Hospital, Barts Health NHS Trust, London, GBR

**Keywords:** free open access, mind the bleep, online medical education (ome), resident doctors, surgical-education

## Abstract

Introduction

Mind The Bleep (MTB) is an online medical education organisation run by United Kingdom-based resident doctors. It represents one of a number of free-open access online medical resources (FOAMed) that have increased in popularity, especially since the COVID-19 pandemic. The surgical team of MTB works to produce high-quality surgical educational resources aimed at early postgraduate doctors. This paper provides an appraisal of the teams' current methods of teaching and the learning points identified from these.

Methods

This was a retrospective mixed-method review analysing quantitative and qualitative data sources including website analytics and feedback surveys. Wilcoxon signed-rank tests were used to identify significant differences between pre- and post-session confidence levels in the subject matter.

Results

A total of 22 online targeted education programmes, with a total of 140 webinars were completed between November 2021 and August 2024. The total live viewership was 6733 participants with a mean of 48.1 per webinar. The total attendance including live and watched later was 10221 participants, with a mean of 73 total viewers per webinar. Of the total viewers, 4714 provided feedback (46.1%). Across all webinar series, we identified a statistically significant increase in participant-rated confidence (p<0.05).

Discussion

Our ability to produce consistent high-quality content is due to leveraging a near-peer teaching model and recruiting resident doctors as teachers. This method ensures cognitive congruence between participant and educator and provides doctors an opportunity to gain teaching experience. Our social media advertising has ensured wide reach. To the best of our knowledge, we provide the first large-scale breakdown of the work done by an online surgical education organisation. Limitations include a low feedback rate and feedback limited to Kirkpatrick type 1 learner reaction.

## Introduction

The COVID-19 pandemic accelerated the availability of medical education online. There has been a well-documented rise in free open-access medical education resources (FOAMed), which act to supplement traditional methods of teaching [1]. The use of online resources in surgical education is less well documented, however, its role is becoming increasingly important. Several recent studies highlight the reduction of surgical exposure in the United Kingdom (UK) medical school curriculum [2,3]. In a country with an already diminished surgical workforce, this is a trend of particular concern [4]. Furthermore, current UK surgical trainees report a reduction in clinical experience before progression to Consultancy [5]. As a result, FOAMed resources play a role in bridging this gap in surgical knowledge required for UK-based medical students and resident doctors.

Mind The Bleep (MTB), a non-profit online education organisation run by UK-based resident doctors, has emerged over the past four to five years as a pivotal FOAMed resource [6]. The website has 49,000 users per month and social media with reach of over 30,000 users. The surgical team of MTB, founded in 2020 is dedicated to producing high-quality webinars and articles aimed at doctors within the Foundation Programme (first two years post-graduation in the United Kingdom).

The surgical team is one branch of the MTB online medical education organisation. Individuals are able to join the team by applying on the MTB website. They will then meet with the overall lead for the surgical team (SP) who will help them formulate a plan for topics and speakers for their online education programme or article. The surgical lead will liaise with other leads in MTB, including the webinar lead (AG) and overall lead (AD) to facilitate new education programmes. Individuals proposing new online education programmes have the responsibility of creating a structured plan with the number of sessions, and topics, identifying speakers and ensuring all content is reviewed by a senior trainee or consultant in that specialty (specialty registrar level or above). The team has also recruited speciality-specific leads, so far both in orthopaedics and plastics, who are registrars in these specialities. These individuals are responsible for any education programmes produced in their specialities.

This paper aims to describe and explore the role of the MTB surgical team in surgical education, provide an appraisal of its current methods of teaching and consider learning points for those interested in contributing to the online surgical education community.

## Materials and methods

This is a retrospective mixed-method survey-based study of the MTB surgical team’s educational content.

Inclusion/exclusion criteria

We included data from all webinars and articles completed by the MTB surgical team from its inception until September 2024. We excluded data from any educational ventures run by other teams in MTB.

Quantitative data sources

This included feedback questionnaires from surgical webinars held on the platform MedAll [7]. Surveys could be adjusted based on the individual teacher’s preference. We focussed on the data on pre- and post-participant levels of confidence in the topic using a five-point Likert scale and participant-rated engagement, interest and helpfulness of the content, as these remained the most included variables in all feedback forms (see the table in Appendix 1). The other source of quantitative data was analytics of viewers for webinars and articles. This information was gained from the platform MedAll, where webinars were held live, and the MTB website [8].

Qualitative data sources

This included questions from the feedback questionnaires completed from surgical webinars held on the platform MedAll. We included the questions, 'what went well?' and 'how could this be improved?' as these were standard across all online education programmes.

Statistical analysis

Categorical variables are described as numbers with a percentage. Wilcoxon signed-rank tests were used to identify statistically significant differences between pre- and post- participant-rated confidence levels for each online education programme.

Ethical considerations

This study used anonymous survey-based data and analytics data from users of the MTB website and viewers on MedAll. All individuals who sign up to use MTB agree to a privacy policy stating that their anonymous data may be used for research purposes.

## Results

Webinars

Webinars are run on the platform, MedAll which requires individuals signing up to confirm their status as a healthcare professional or university student. Webinars are advertised on relevant social media pages, including the MTB Facebook groups for each geographical deanery, Twitter, Instagram, and the MTB monthly newsletter.

In total, the MTB surgical team has produced 140 webinars, across 22 online education programmes, over three years from November 2021 to September 2024 (Table 1). The mean number of webinars per online education programme was 6.36, with a range of 2-12 webinars per programme. The total live viewership was 6733 participants. The total overall viewership including those who watched the recorded sessions later was 10,221 participants, with an overall mean of 73 total viewers per webinar. Feedback was completed by 4714 participants, 46.1% of the total attendees.

**Table 1:** Summary of online education programmes NA: Not available, ENT: Ear, nose & throat, MRCS: Membership of the Royal College of Surgeons, DO-HNS: Diploma in Otolaryngology-Head and Neck Surgery, CST: Core surgical training

Teaching Programme	Number of Webinars	Number of Live Viewers	Number of participant feedback questionnaires completed	Total number of viewers
Urology series	11	1217	1388	2152
Surgical admissions/on take	3	NA	80	315
Ophthalmology	6	278	301	410
Orthopaedics series 1	8	522	506	657
Vascular	10	648	347	657
Surgical cases	6	329	165	329
ENT series 1	6	233	170	413
MRCS series 1	12	962	460	962
Plastics series 1	4	408	169	577
Anatomy series	5	192	97	288
Women in Surgery	7	100	16	162
Peri-operative management	4	112	67	112
Surgical oncology	6	136	108	136
MRCS series 2	5	214	131	293
DO-HNS series	2	31	22	31
Neurosurgery	7	238	100	258
On call series	6	164	109	164
CST interview series	7	302	117	754
Plastics series 2	3	64	31	70
Prescribing in surgery	9	273	150	812
ENT series 2	8	167	116	423
Orthopaedics series 2	5	143	64	246

Confidence levels

Pre- and post-webinar participant-rated confidence levels were recorded for 20 of the 22 online education programmes. There was a statistically significant increase in participant confidence levels seen across all programmes (Table 2).

**Table 2:** Results of Wilcoxon signed rank test analysis for each online education programme ENT: Ear, nose & throat, MRCS: Membership of the Royal College of Surgeons, DO-HNS: Diploma in Otolaryngology-Head and Neck Surgery, CST: Core surgical training

Teaching Programme	Mean confidence level pre-series	Mean confidence level post-series	Wilcoxon signed rank test Z Score	P value
Surgical admissions/on take	2.98	4.27	- 13.89	p<0.001
Ophthalmology	2.83	4.23	- 5.578	p<0.001
Orthopaedics series 1	2.94	4.03	- 16.02	p<0.001
Vascular	2.83	4.05	- 13.85	p<0.001
Surgical cases	2.81	4.08	- 9.84	p<0.001
ENT series 1	2.74	3.98	- 10.24	p<0.001
MRCS series 1	2.73	3.90	- 16.91	p<0.001
Plastics series 1	2.77	4.18	- 13.01	p<0.001
Anatomy series	2.77	3.93	- 7.73	p<0.001
Peri-operative management	3.12	3.91	- 3.90	p<0.001
Surgical oncology	3.01	4.21	- 7.76	p<0.001
MRCS series 2	2.89	4.10	- 8.93	p<0.001
DO-HNS series	2.68	3.95	- 3.95	p<0.001
Neurosurgery	3.12	4.24	- 7.46	p<0.001
On call series	3.10	4.48	- 8.23	p<0.001
CST interview series	2.72	4.12	- 8.83	p<0.001
Plastics series 2	2.69	4.13	- 3.10	p<0.001
Prescribing in surgery	2.94	4.13	- 9.15	p<0.001
ENT series 2	3.24	4.29	- 6.40	p<0.001
Orthopaedics series 2	3.17	4.25	- 5.85	p<0.001

Engagement, helpfulness and interest

As part of the feedback survey, participants were asked to rate the webinars using a five-point Likert scale based on how engaging they found the session, how helpful the session was at improving their understanding and their clinical practice and how interesting they found the content.

Across all online education programmes, the interest and helpfulness ratings were equal to or greater than four. All online education programmes apart from one had an engagement rating of equal to or greater than four. The results were available for 19 of the 22 webinar programmes (Table 3).

**Table 3:** Summary of the engagement, interest and helpfulness mean ratings across online teaching programmes NA: Not available, ENT: Ear, nose & throat, MRCS: Membership of the Royal College of Surgeons, DO-HNS: Diploma in Otolaryngology-Head and Neck Surgery, CST: Core surgical training

Teaching Programme	Engagement mean rating	Interest mean rating	Helpfulness mean rating
Surgical admissions/on take	4.33	4.50	NA
Orthopaedics series 1	4.125	NA	4.375
Vascular	3.94	4.25	4.125
Surgical cases	4.17	4.42	4.33
ENT series 1	4.25	4.25	4.25
Plastics series 1	4.125	4.375	4.375
Anatomy series	4.10	4.40	4.10
Women in Surgery	4.42	4.42	4.50
Peri-operative management	4.125	4.50	4.25
Surgical oncology	4.20	4.50	4.50
MRCS series 2	4.10	4.30	4.50
DO-HNS series	4.00	4.25	4.25
Neurosurgery	4.21	4.42	4.50
On call series	4.50	4.50	4.50
CST interview series	4.25	4.50	4.20
Plastics series 2	4.20	4.50	4.25
Prescribing in surgery	4.22	4.22	4.20
ENT series 2	4.25	4.44	4.44
Orthopaedics series 2	4.10	4.20	4.10

Qualitative feedback

Qualitative feedback data was available for 20 of the 22 online education programmes. A thematic analysis from the qualitative feedback gained from all webinars was performed. Key themes from each programme were picked out and included in Table 4.

**Table 4:** Thematic analysis results for each online education programme ENT: Ear, nose & throat, MRCS: Membership of the Royal College of Surgeons, DO-HNS: Diploma in Otolaryngology-Head and Neck Surgery, CST: Core surgical training

Education Programme	What went well?	What could have been better?
Surgical admissions/on take	1) Conciseness of presentations 2) Explanation of concepts 3) Use of questions for the audience 4) Use of case examples	1) More time needed for each session 2) More interactivity
Orthopaedics series 1	1) Interactivity 2) Explanation of concepts 3) Use of images 4) Use of cases 5) Overall presenting skills	1) Technical issues 2) More interactivity
Vascular	1) Use of interactive quiz 2) Use of images 3) Explanation of concepts 4) Overall presenting skills	1) More interactivity 2) Technical issues 3) More clinical cases
Surgical cases	1) Use of clinical cases 2) Audience participation 3) Explanation of concepts 4) Overall presenting skills	1) More time allocated to webinars 2) More images needed 3) More interactivity
ENT series 1	1) Use of clinical cases 2) Explanation of concepts 3) Overall presenting skills 4) Revision of anatomy prior to explaining surgical management	1) More images 2) More cases required 3) Length of session could be longer 4) Explanations to be written as well
MRCS series 1	1) Good memory aids 2) Focus on high-yield question areas 3) Interactivity 4) Relevance of content to examination	1) More practice questions 2) Technical issues 3) Presentations were rushed
Plastics series 1	1) Good use of images 2) Explanation of concepts 3) Overall presenting skills	1) Technical issues 2) More detailed presentation for burns topic
Anatomy series	1) Overall presenting skills 2) Explanation of concepts 3) Wide range of topics covered 4) Level of detail 5) Use of images	1) More interactivity 2) More time per presentation
Woman in Surgery	1) Enjoyable and inspiring 2) Helpful information	1) More time per presentation
Peri-operative management	1) Overall presenting skills 2) Relevance of content 3) Use of cases	1) More interactivity 2) Use of summary slides 3) Technical issues
Surgical oncology	1) Overall presenting skills 2) Explanation of concepts 3) Conciseness of presentations	1) More interactivity 2) More images 3) Too much information per webinar
MRCS series 2	1) Explanation of concepts 2) Overall presenting skills 3) Use of questions	1) Technical issues 2) Poor organisation regarding timings of presentations
DO-HNS series	1) Good use of images 2) Thorough coverage of topics	1) More interactivity
Neurosurgery	1) Comprehensive coverage of key topics 2) Use of interactive polls 3) Overall presenting skills 4) Use of cases	1) More focus required on application process 2) Technical issues 3) Provision of reading materials
On call series	1) Good interactivity 2) Use of cases 3) Overall presenting skills	1) Technical issues 2) More images 3) Pacing too fast
CST interview series	1) Good interactivity 2) Practical advice about how to answer interview questions 3) Use of real-life examples	1) More sessions and increased length of sessions
Plastics series 2	1) Key information required for emergencies 2) Overall presenting skills	1) More sessions
Prescribing in surgery	1) Good interactivity 2) Use of clinical cases 3) Information presented pitched at the right level for foundation doctors	1) Technical issues 2) More sessions and Increased length of sessions
ENT series 2	1) Overall presenting skills 2) Comprehensive coverage of key ENT topics	1) More interactivity
Orthopaedics series 2	1) Overall presenting skills 2) Good interactivity 3) Use of concise summaries	1) Less text on slides 2) Technical issues

Articles

Articles are published on the MTB website under the specific speciality pages. Article authors are required to have the content reviewed by a senior trainee or consultant in that specific speciality. The MTB surgical team has published 99 articles, across nine surgical specialties (Table 5). Article viewers can rate the article out of five and can leave comments, which can be replied to by either member of the MTB team or other users.

**Table 5:** Summary of the articles produced across each surgical specialty ENT: Ear, nose & throat

Specialty	Number of articles	Total number of article views	Mean rating
ENT	9	7817	4.86
General Surgery	18	17861	4.62
Obstetrics & Gynaecology	6	6407	4.75
Ophthalmology	42	19519	4.85
Orthopaedics	8	4260	4.76
Maxillofacial Surgery	9	3660	4.82
Plastic Surgery	2	1103	5
Urology	4	4683	4.70
Vascular	1	1206	5

## Discussion

To the best of our knowledge, this retrospective review of the MTB surgical team represents the first in-depth evaluation of an online surgical education organisation’s practices and outcomes in the literature. Over three years, the MTB surgical team’s 140 webinars and 99 articles were viewed by over 10,000 participants with positive feedback and statistically significant improvements in participant confidence across all online education programmes.

In the thematic analysis, lack of interactivity was a feedback point identified in 9/20 (45%) of the education programmes. Interactivity as an effective teaching tool is underpinned in cognitive and constructivism learning theory and by several studies in the literature [9-11]. Ensuring future series focus on interactivity in the form of audience participation, case discussions and polls will help to address this limitation and hopefully lead to more effective learning.

Our overall output and positive feedback highlight the strength of our near-peer teaching (NPT) model. PT is defined as a learning model where the teacher is at least one year senior to the learner [12]. The majority of individuals recruited who produced these online education programmes were at the level of foundation doctor or core trainee. There are several proposed benefits to the NPT learning method. Teachers are more likely to communicate and explain teaching points in a language similar to learners, a phenomenon known as cognitive congruence [13]. Teachers, having recently undergone similar training, have a unique insight into the specific needs and challenges faced by learners. This approach is particularly advantageous in teaching surgical topics to foundation doctors when there is no standardised national curriculum for early postgraduate surgical education. Near-peer tutors create a relatable and safe learning environment, encouraging active participation and questions.

A 2021 literature review and meta-analysis by Khapre et al. investigated the effectiveness of NPT in the education of undergraduate medical students. The authors did not identify a statistically significant difference between NPT groups and expert teaching groups across the sixteen studies included. Although these results are encouraging, the studies showed considerable heterogeneity across populations, study design, size and methodology. The use of NPT for enhancing surgical education has been described by a number of papers in the literature, with the majority highlighting the positive impact of NPT on technical skills training, including knot tying, suturing and laparoscopy [14-17]. Musbahi et al. investigated the use of junior doctors as near-peer tutors, focusing on key surgical topics relevant to medical school examinations. The study albeit small, with a participant number of 53 students, identified a significant increase in participant confidence in the topic after the teaching session and an increase in knowledge, based on pre- and post-programme test results [18].

The benefits of these near-peer online teaching ventures are not limited to the students but also extend to the teachers. Teaching is a reflective practice. It requires the teacher to evaluate their understanding of a subject, identify gaps that need to be addressed and formulate a way to convey their knowledge to their students. The role of reflection as a learning tool for doctors has been extensively investigated in the literature. A 2017 literature review by Winkel et al. examined 16 studies investigating reflection as a teaching tool for doctors in training. The authors identified the use of reflection in engagement in learning, the learning of complex subjects and empathy [19]. For doctors applying for further specialty training, teaching is looked upon favourably and scores highly in the application processes. Working within the MTB organisation provides doctors with a network of like-minded individuals from different specialities to exchange ideas and seek advice from. Individuals also have opportunities to subsequently apply for leadership roles within the organisation.

A significant contributing factor in our ability to reach a large audience has been the use of free-to-access digital platforms. Educational content is advertised on social media platforms including Facebook, Instagram and Twitter. Webinars can be watched live or later on our MedAll and YouTube pages. Articles are published on our website. The digital age of medical education has been investigated especially during the COVID pandemic, where social distancing rules necessitated a shift from offline to online learning. Studies comparing these two methods found comparable outcomes [20]. Furthermore, new pedagogical learning theories have been described to better understand and manage this evolution [21]. One of the most cited is the theory of connectivism, where knowledge is gained by joining and interacting in a community with the use of technology [22].

Practically, the most obvious advantage of the digital space for education is the ability for learners to interact with experts/teachers without concern for logistics and time. Furthermore, it has been postulated in the literature that the online space allows learners to develop their skills of self-regulated learning [23]. As doctors are required to be lifelong learners, this skill needs to be practiced and honed. Indeed, in the UK in particular, a significant number of medical schools use ‘problem-based learning.’ A teaching format of self-regulated learning where a learning scenario is presented that requires students to identify their learning objectives which they are then able to address in their own time. Access to the digital space and the medical educational content it provides allows users to meet this need.

There are a few examples of online surgical education ventures aimed at foundation doctors in the literature. Kam et al. describe a national online teaching programme for foundation doctors focussed on surgery, in affiliation with the Royal College of Surgeons of Edinburgh. Ten sessions with a total of 131 participants were held on Zoom. About 95.2% of participants responded that they felt more confident in the topic post the session. The study was limited by the small sample of participants, especially in the context that the programme was national [24]. Lee and Ahmed described a series of six virtual lectures delivered on Zoom, aimed at Foundation doctors covering neurosurgery [25]. The authors reported 31 participants. They identified significantly improved participant confidence levels and post-lecture test scores. This venture was focused on a single centre with a small number of participants.

There are also more large-scale online surgical education ventures run by more established surgical societies and organisations. The Royal College of Surgeons of England provides online modules as part of their Postgraduate Certification in Surgery; however this comes with a fee and as a result, it is not as easily accessible to all foundation doctors [26]. The Association of Surgeons in Training (ASiT) is a UK-based independent body representing the views of surgical trainees across all specialties [27]. Members pay a yearly fee, but with this comes numerous benefits including discounts to events, access to educational content, access to journals, ability to apply for prizes and other benefits. The Black Belt Academy of Surgical Skills is a free online resource designed to help doctors with an interest in surgery practice their basic surgical skills at home with minimal equipment [28]. Most similar to MTB, is the ‘teachmeseries.’ This is an online collection of educational resources, which includes the ‘Teach Me Surgery’ website [29]. The website contains articles encompassing topics on all surgical specialities aimed at the early postgraduate level. Similar to MTB the venture is run by doctors in training, with individuals being able to provide feedback on articles. There is no accessible data available on any outcome measures or practices for the above examples.

Limitations

Although feedback is required in order to be eligible for a certificate of attendance to our webinars, overall feedback was gained from only 46.1% of participants, and this may not be representative of the views of the entire cohort. Furthermore, our feedback results are limited to Kirkpatrick type 1 learner reaction [30]. To more accurately evaluate our work, focus on knowledge retention and use in clinical practice is needed. Secondly, although our advertising targets foundation doctors, and on our social media pages this cohort represents the largest percentage of our audience, we do not collect data on individual audience members’ levels of training or occupations and therefore there will be feedback data included from medical students as well as allied healthcare professionals, that does bias the overall conclusions. Furthermore, our data collection strategy has been retrospective and therefore is at risk of reporting bias.

Implications for future research

Our paper can be used as a framework from which other online surgical education organisations can build to report their results. An increase in the literature in this area, would help build an evidence base and hence inform future practice. Future work with a prospective data collection strategy would ensure a reduction in the risk of bias encountered from missing data.

Future work could be done to survey and identify the educational requirements of foundation doctors completing surgical rotations. A focus on outcomes related to Kirkpatrick levels two to four would help more accurately discern an online organisation's impact on knowledge gain and the resultant effect on clinical practice.

## Conclusions

This retrospective review of the MTB surgical team’s practices over the past three years has highlighted the effectiveness of our approach in delivering high-quality free educational resources to a large audience. Our findings emphasise the need for continued surgical educational content for foundation doctors. The lack of interactivity has been identified as a learning point to improve our future resources, with the aim of enhancing comprehension and knowledge retention.

Our work serves as an important framework that can be used by other education organisations. Future research into practices by other organisations would help enhance the literature and inform online education strategies. A focus on outcomes related to knowledge acquisition, retention and translation to clinical practice would more accurately determine the impact of our work.

## Appendices

**Table 6:** Example of feedback form questionnaire from a teaching programme

Feedback Questions	1	2	3	4	5
How confident were you in this topic before the teaching session?					
How confident are you in this topic after the teaching session?					
How engaging did you find the teaching?					
How helpful was the content?					
How interesting did you find the format?					
What Went Well?					
What could have been better?					
